# Retraction: Evaluation of carboxymethylated *plectranthus edulis* starch as a suspending agent in metronidazole benzoate suspension formulations

**DOI:** 10.1371/journal.pone.0254624

**Published:** 2021-09-07

**Authors:** Yonas Brhane

The author retracts this article [1] because it reported material from [2], published in 2019 by the Pharma Book Syndicate (a unit of BSP Books Pvt. Ltd.) which is not offered under a Creative Commons Attribution License. The retracted *PLOS ONE* article was removed from the *PLOS ONE* website at the time of retraction and the removed contents are no longer offered under the Creative Commons Attribution License. Readers should refer to [2] for the copyright notice.

## References

[1] Brhane Y (2020) Evaluation of carboxymethylated *plectranthus edulis* starch as a suspending agent in metronidazole benzoate suspension formulations. PLoS ONE 15(3): e0228547. 10.1371/journal.pone.0228547 32119679 PMC7051080

[2] Brhane Y (2019) Evaluation of Carboxymethylated Plectranthus edulis Starch as Suspending Agent in Metronidazole Benzoate Suspension Formulation. Int J Pharm Sci Nanotech 12(5): 4672–4680.

